# The Social Consequences of Infertility among Iranian Women: A Qualitative Study

**DOI:** 10.22074/ijfs.2015.4181

**Published:** 2015-02-07

**Authors:** Syedeh Batool Hasanpoor-Azghdy, Masoumeh Simbar, Abouali Vedadhir

**Affiliations:** 1 Department of Reproductive Health and Midwifery, Faculty of Nursing and Midwifery, Iran University of Medical Science, Tehran, Iran; 2 The Research Center for Safe Motherhood , Department of Reproductive Health and Midwifery, Faculty of Nursing and Midwifery, Shahid Beheshti Medical Sciences University, Tehran, Iran; 3 Department of Anthropology, Faculty of Social Sciences, University of Tehran, Tehran, Iran

**Keywords:** Infertility, Violence, Divorce, Social Isolation, Social Exclusion

## Abstract

**Background:**

Infertility may prevent couples to achieve the desired social roles and lead to some social and psychological problems. This study aimed to explain the social consequences of infertility in Iranian women seeking treatment.

**Materials and Methods:**

A qualitative content analysis was conducted based on 32 semi-structured interviews with 25 women affected by primary and secondary infertility with no surviving children. The participants were purposefully selected with maximum variability from a fertility health research center in Tehran, Iran, from January to October 2012. Data were collected using semi-structured interviews and analyzed using the conventional content analysis method.

**Results:**

Our findings indicate that the consequences of infertility are divided into five main categories: 1. violence including psychological violence and domestic physical violence, 2. marital instability or uncertainty, 3. social isolation including avoiding certain people or certain social events and self-imposed isolation from family and friends, 4. social exclusion and partial deprivation including being disregarded by family members and relatives and reducing social interactions with the infertile woman and 5. social alienation.

**Conclusion:**

This study reveals that Iranian women with fertility issues seeking treatment face several social problems that could have devastating effects on the quality of their lives. It is, therefore, recommended that, in Iran, infertility is only considered as a biomedical issue of a couple and pay further attention to its sociocultural dimensions and consequences.

## Introduction

Infertility could be a life crisis with a wide range of sociocultural, emotional, physical and financial problems (1, 2). Approximately 8-12% of all couples are infertile worldwide, indicating one out of ten couples experiences primary or secondary infertility (3, 4). A widespread study was conducted in 2005 to determine the prevalence of infertility in Iran. This study showed that about a quarter (24.9%) of Iranian couples have experienced primary infertility at some stage of their marital life (5).

In developing countries, children are highly valued for social, cultural and economic reasons (6). Many religions and faiths put a great emphasis on fertility and childbearing. In Islam, the position of motherhood is highly honored and it is widely believed among Muslims that "Heaven lies at the feet of mothers" (7). Reproduction is highly recommended in Christianity as well, but infertility is considered another blessing from God by some Christians (8). Judaism encourages its followers to procreate and some Jewish scholars allow using artificial means for this purpose (9).

The infertile couple may suffer from social pressures in addition to the direct impacts of infertility (4). Infertility could be a source of social and psychological suffering for women in particular. In some communities, the childbearing inability is only attributed to women, hence there is a genderrelated bias when it comes to a couple’s infertility (6, 10). Existing research has suggested that infertility affects women more deeply than men (11). Infertile women may experience domestic violence, economic deprivation, social isolation, loss of social status and ostracized marital lives (4, 10, 12). As a result, a private pain, namely infertility, could turn into a public and unpleasant stigma with complex and devastating consequences (4).

Even in the case of treatments like gamete donation, the risk of social impacts could not be ignored. A research has showed how Iranian women coaxed or coerced into accepting third-party gamete donation may suffer from consequences, including emotional and physical abuse, abandonment, and divorce (13). Abbasi-Shavazi et al. reported that gamete and embryo donation may result in social stigma in the community (14). In Iran, like most of the developing countries, childbearing is considered as a social valuable and a necessary condition for married women. In this setting, childlessness and infertility are commonly considered as unpleasant features for couples. The term "cold stove" refers to families without children in the Iranian community. These norms are deeply rooted in the belief system of the people and are supported by religious and traditional views. Criticality of the childlessness is also evident in the Iranian Family Protection Law. According to the article 9 of this law, infertility can legally and religiously justify the termination of a marriage by divorce (15).

In the Iranian culture, the patriarchal beliefs for the necessity of reproduction, lack of social and economic support for many women, slim chance of remarriage for infertile women and social disapproval of single life are some factors that may intensify the psychological sufferings of infertile women (16). Therefore, this study focuses on infertile "women".

A thorough study of the social consequences of infertility in women in the Middle East is of particular importance where a woman’s social status, her dignity and self-esteem are closely related to her procreation potential in the family and in the society as a whole (4). Any plan to support and to empower the infertile women in order to face this challenge should be based on a comprehensive understanding of the various consequences of this issue in the society. Knowledge of social and psychological consequences of infertility would guide the public policies and the social sector programs (2, 17, 18).

In addition, review of the literature has revealed that infertility does not take place in a socio-cultural vacuum. The socio-cultural context in which the infertile couple living affects all aspects related to infertility (19, 20). Nevertheless, studies of fertility in developing countries are in most cases done with a focus on the biomedical, moral or psychological aspects of the issue with less attention to the sociocultural and political context (21).

Since the cross-sectional quantitative studies are still common in dealing with the social and psychological consequences of infertility, regardless of their inadequacies in sorting out cause and effect (2), this study was designed and conducted qualitatively to examine the social consequences of infertility in Iran. The value of a qualitative methodology in the evaluation of the implications of infertility has been increasingly recognized (22). This approach avoids the more rigid format for gathering quantitative data; a format which limits the ability of the participants to reply and prevents the exploration of unexpected topics (23). More specifically, this qualitative study aims to reveal the experiences and understanding of Iranian infertile women seeking treatment from various social consequences of the infertility.

## Materials and Methods

To explain the experiences of Iranian infertile women seeking treatment with a focus on the social consequences of infertility, a qualitative approach was taken in this study. A type of qualitative content analysis (QCA), i.e. the Conventional Qualitative Content Analysis (CQCA), was drawn on to manage and to analyze data gathered from the participants who were women with primary and secondary infertility with no surviving children. The study setting was the Vali-e-Asr Fertility Health Research Center in Tehran, where about 1500 infertile women from different parts of the country are seen annually. Some women were referred to the center by family doctors and primary care physicians, while others generally learned about the center through their friends and relatives. Treatment costs were not covered by insurance, but as being a government-funded center, a part of treatment expenses was subsidized. The total costs for *in vitro* fertilization (IVF) and intrauterine insemination (IUI) treatments were roughly $1,250 and $100 in USD, respectively, at the time of data collection. The sampling procedure was done using a purposeful sampling strategy and the interviews continued until data saturation. So a total of 23 participants, plus two additional participants, were included. The inclusion criteria consisted of female infertility, absence of chronic diseases and mental illnesses, no adopted children and willingness to participate in the study.

The characteristics of participants in the study sample with maximum variance assured the quality of the study by intensifying the validity and transferability of findings (24).

An informed consent was obtained from all participants after explaining the study in detail and the need to obtain audio recording of the interviews by main investigator. It was also emphasized that participation in this study was voluntary, and strict adherence to confidentiality rules regarding personal information was guaranteed. Prior approval for the study was obtained from the Ethics Committee of Shahid Beheshti University of Medical Sciences.

Data was collected using the semi-structured interviews, observations, field notes and recorded voices. Throughout the interviews, attention was paid to the non-verbal behaviors of the participants. Interviews ranged from 60 to 90 minutes with a frequency of one to two per each participant. Overall, 32 interviews were conducted with 25 participants. All interviews were conducted in Farsi. Recorded interviews were listened, transcribed verbatim and analyzed by the investigator. Observations regarding a participant’s demeanor, reactions and facial expressions in various parts of the infertility center, such as the reception area, waiting room, examination and sonography rooms, were also recorded. The field notes were properly recorded and immediately analyzed in detail. In terms of timing, the data collection and analysis lasted from January to October 2012.

As mentioned previously, data analysis was done using the CQCA. In this mode of qualitative data analysis, the systematic classification processes are drawn on to identify codes and themes within the content of the study. In addition, codes are extracted from the meaningful units of the participants’ descriptions and are classified with reference to similarities or dissimilarities, based on the relevant themes identified by the CQCA (25, 26).

Several measures were taken to strengthen and to distribute credibility of the data collection process, analysis and results. These included assuring the adequate diversity of participants in terms of socio-demographic features, increasing contact time with the participants and the research setting, clarifying the objectives of the study for the participants, and analyzing transcriptions immediately after the interview and getting feedback for the next interview. All data and evidences were checked, corrected and revised using the recorded voices and reactions of the participants. To examine the transferability of the study, data were made available to some infertile women who did not participate in the study, asking them to compare the results with their own experiences (24).

## Results

A total of 25 women between the ages of 21-48 with a history of primary or secondary infertility with no surviving children were interviewed. One of them was illiterate and the other’s education ranged from elementary to Ph.D. degree. Duration of marriage and infertility treatment ranged from 3-22 years and 1-14 years, respectively. Two of them had more than one decade of experience in seeking and in receiving infertility treatment. The main characteristics of the recruited infertile women with a focus on age, education, occupation, family income, marital status, duration of infertility, duration of infertility treatment and type of treatment are shown in table 1. The main extracted concepts are included in the five main categories and sub-categories by means of the CQCA, as shown in table 2.

**Table 1:** The characteristics of the participants in this study


NP	Age (Y)	Education	Occupation	Family income ($)	Duration ofmarriage(Y)	Typeof infertility	Durationof infertility(Y)	Durationof treatment(Y)	Typeof treatment

**P1**	21	High school diploma	Housewife	900	3	Secondary	2	2	M + IUI
**P2**	31	High school diploma	Housewife	326	7	Primary	6	6	M + IVF
**P3**	31	B.Sc.	Employee	1.305	3	Secondary	2	2	M + IUI
**P4**	30	High school	Housewife	408	10	Secondary	7	6	M + IVF
**P5**	43	Primary school	Housewife	408	22	Primary	14	7	M + IVF
**P6**	33	Ph.D.	Employee	1.060	10	Secondary	2	2	M + IVF
**P7**	25	B.Sc.	Employee	1225	5	Primary	5	2	IVF
**P8**	35	Illiterate	Housewife	228	5	Primary	3	3	M
**P9**	39	B.Sc.	Employee	2039	3	Primary	2	2	M + IUI + IVF
**P10**	24	Middle school	Housewife	163	10	Primary	8	8	M + IUI + IVF
**P11**	34	Primary school	Housewife	815	8	Primary	7	7	M + IVF
**P12**	27	High school	Housewife	285	7	Primary	6	6	M + IUI
**P13**	23	High school diploma	Housewife	285	2	Primary	1	1	IVF
**P14**	29	Middle school	Housewife	653	5	Primary	3	3	M + IUI
**P15**	36	B.Sc.	Employee	1060	3	Primary	2	2	M + IVF
**P16**	30	High school	Housewife	571	7	Secondary	2.5	2.5	M + IVF
**P17**	26	B.Sc.	Employee	652	3	Primary	2	2	M + IUI
**P18**	28	Middle school	Housewife	570	3	Primary	2	1	M + IUI
**P19**	27	High school diploma	Housewife	653	4.5	Primary	2.5	2.5	M + IUI
**P20**	31	Middle school	Housewife	326	13	Primary	12	12	M + IUI
**P21**	37	High school diploma	Employee	815	13	Secondary	12	5	M + IUI
**P22**	35	Primary school	Housewife	408	15	Primary	14	14	M + IUI + IVF
**P23**	29	Master’s degree	Employee	2.039	6	Secondary	4	4	M + IUI
**P24**	22	Middle school	Housewife	224	8	Primary	6	1	M + IUI
**P25**	48	Middle school	Retired	815	7	Primary	6	6	M + IVF


NP; Number of participants, M; Medicinal, IUI; Intrauterine insemination and IVF; In vitro fertilization.

**Table 2:** The main categories and their sub-categories in this study


The main categories	Sub-categories

**Violence**	Psychological violence and domestic physical violence
**Marital instability or uncertainty**	
Social isolation	Avoiding certain people or certain social events and self-imposed isolationof social interactions with family and friends
Sense of social exclusion and relative deprivation	Being disregarded by family members and relatives and reducingsocial interactions with the infertile woman
Social alienation	-


### Violence

This category includes two sub-categories: psychological violence and domestic physical violence.

The first sub-category, the psychological violence, is usually experienced as the form of stigma. In a broad conceptualization, stigma as a multifaceted phenomenon is the result of a process in which a series of interrelated components combine to generate stigma. Stigma is also determined as a characteristic of persons that is unlike to a norm of a social unit, while norm is defined as a certain way that a person is supposed to behave at a certain time. Stigmatized individuals possess some attribute, or characteristic, that conveys a social identity that is devalued in a particular social context. Stigma can be seen as a relationship between an attribute (mark) and a stereotype (undesirable characteristics). Hence, the stigmatized people experience discrimination and loss of status. When people are labeled, set apart, and linked to undesirable characteristics, a rationale is constructed for devaluing, rejecting, excluding and de-authorizing them (27, 28).

Most infertile people who had experienced stigma felt the loss of dignity and social status by the spouse, significant others, family members and community. They reported experiences of blaming and inattention by others and sense of humiliation for being infertile. Some participants also spoke about the discrimination they experienced from their in-laws. Participants with lower education level and lower family income were subjected to more psychological violence by their husband. A participant said, "My husband frequently makes wisecracks about my infertility by remarking that my brother, for example, has married later than us, but he has a child now" (Participant 8).

Another participant with a shaky voice and tears said, "My husband told me that I was barren because I married too late" (Participant 25).

Nearly all participants of the study had suffered from social pressure, directed mostly by close relatives and in-laws, for their lack of parenthood. In the same context, a participant noted, "There is a common proverb here that an infertile woman looks like a dried tree" (Participant 10).

Social pressure stigma was higher in participants who lived in rural areas with low socio-economic level.

A participant who lived in a village said, "I paid twice the amount of 320 dollars for my medications, but the rumor spread in the village that I had spent 2500 dollars for the medications, but still couldn’t have children" (Participant 5).

The use of some treatments such as oocyte donation or surrogacy caused a stigma for some participants among family and friends; therefore, the participants tried to hide their treatment methods from others. One participant said, "Only my mother knows about my treatment method. I don’t let other people know about this because later they will talk behind my back. They will say her child’s father is someone else, mother is also someone else. This [treatment] is not accepted in our culture yet" (Participant 7).

The participants shared their experiences about stigma resulting from certain actions and words of the relatives and people around in some social events. For example, they pointed to the people’s pitiful gaze, discriminating behavior, and engagement in public prayers and traditional ceremonies in hope to resolve their infertility problem. As one of the infertile women expressed,"The woman, who prays regularly for the prosperity and health of people at the end of praying ceremony, prays for me to carry a baby by the next year. When she did this in public, I experienced a strong sense of inferiority" (Participant 19).

An infertile woman said, "I hate people when they look at me with pity" (Participant 16).

About 1/3 of infertile participants were harassed with the verbal violence by their husbands. Most of them expressed that when their husbands began to insult them, they had to stay quite. One of the participants (Participant 2) stated that the verbal violence between couples often took place when they were seeking treatment for their infertility.

Two of the participants experienced domestic physical violence as a result of their infertility in addition to the psychological violence. Both of these participants had low levels of education and family income. These two women reported pulling hair, slapping and pushing around by their husbands. As one of the participants narrated her experience with a broken voice and with tearful eyes,"My husband pulled out my hair and slapped me in my face. He also threw me out of our home. I repeatedly called him because I didn’t want to destroy our marriage" (Participant 20).

In cases where the husband and family of the infertile woman believed that having or not having children was God’s will, less physical violence and stigma were reported by the participants, even for low socio-economic level families.

### Marital instability or uncertainty

The majority of participants in this study stated that if their husbands wanted to remarry, they would separate from the spouse. According to the infertile women, there are some factors contributing to the possibility of divorce among the infertile couples. These influencing factors are as follows: "the high social pressure for remarriage of husband by the relatives and/ or people around", "husband’s decision to remarry", "repeated treatment related infertility", and "lack of proper understanding by husbands of the social and psychological pressures experienced by their wives." As one of the participants expressed, "From all sides, people recommend my husband to get remarried. His family, the relatives and significant others instruct my husband to divorce me, to look for and to remarry a fertile woman" (Participant 20).

A participant who was aware of her infertility problem before the marriage stated, "Although I had explained my situation to my husband and he accepted it, I still fear for my future. If the medications don’t work, I am not sure that my marriage would last" (Participant 7).

A good number of participants pointed out that although their husbands were encouraged or pressured by the relatives to remarry, their husbands seemed eager to accept the prospect in his current marital life. However, of all participants, no one accepted this state of affairs. As a participant stated, "I explicitly told my husband that if he provides the expenses and cooperates with me during infertility treatment, such as the IVF, and that if I can’t be pregnant, I would voluntarily facilitate his remarriage. While doing this, I would get a divorce because I cannot tolerate sharing my marriage with another woman, but my husband replied that he would not want me out of his life" (Participant 14).

### Social isolation

This category also consists of two sub-categories: avoiding certain people or certain social events and self-imposed isolation from social interactions with family and friends. The findings of this study revealed that the majority of infertile women chose to avoid dealing with socio-psychological stress of infertility. This means that they preferred to employ a maladaptive coping mechanism, characterized by the continuous effort to protect themselves from social and psychological harm (29, 30). They operationalized this coping strategy in various ways including modifying or eliminating the conditions that gave rise to the problem. For example, some women stayed away from children, pregnant women, infertile peers and refused to watch television programs concerning fertility and infertility. As an infertile women narrated, "When I see a pregnant woman, I say to myself, lucky her. In this situation, I am so sad. This is why I constantly stay away from pregnant women" (Participant 3).

The infertile women also preferred to be absent from some social ceremonies and events, while they avoided contact with those who criticize them. This is, therefore, a coping strategy to manage the stigma of infertility and to get rid of social pressure of people around. An infertile woman expressed her coping mechanism in this way, "I do not like to attend the family gatherings. If I have to be there, I try to be busy with cooking in kitchen or any place far from the folks. As a rule, I do not like to join the social events and gatherings" (Participant 4).

Furthermore, the participants sometimes preferred to reduce interactions with curious people around due to the shameful image of the infertility in the society and/or due to avoidance of their offensive questions. Moreover, some participants tried to reduce their communication and interactions with all to prevent their husbands’ discomfort or to hide the issue of infertility. As a participant remarked, "When my husband and I go to someone’s house, he says that he is embarrassed when someone looks at me or says things with pity. Considering the point that my husband is so sensitive to these looks and words, I hardly want to visit relatives. Otherwise, it does not matter to me" (Participant 23).

### Feeling of social exclusion and relative deprivation

This category includes two sub-categories: being disregarded by family members and relatives and reducing social interactions with the infertile woman. Participants in some conditions, such as the arrival of new baby to family or presence of a newly pregnant woman at family gatherings, were ignored by family or relatives. As one of the participants expressed her experience and feeling in this way, "Now, there is a new bride in the family, so they make all excuses to ignore me and this is made evident by their behavior and looks" (Participant 8).

Another factor that creates a feeling of relative deprivation and social exclusion for the infertile women is reducing social interactions with the infertile woman by the family members and the friends. According to the participants, the relatives and friends, especially those with children, preferred to socialize with families who had children. One of these women voiced her feelings here, "I feel that the family members are more interested in visiting my sister-inlaw (Jari) as she is productive. They keenly go there, whilst they most reluctantly come to our place and visit us as if we are not family members": (Participants 12).

### The social alienation

Alienation is the process whereby individuals become estranged from the society and elements of culture, which then confront them as an independent, objectified force (22). In this view, the infertile women experiencing alienation suffer from confusion in navigating their social behavior and in adapting certain social norms. This type of social alienation can be characterized by confusion or uncertain behavior in dealing with children and pregnant women and in joining in social or certain religious ceremonies, As a participant said, "If one interacts with kids, folks are looking at her, saying that she yearns to have kids. If one does not interact with kids, folks say that she did not pay attention to kids, as she is very jealous of women becoming mothers. You always wonder how to behave" (Participant 6). Another participant said, "I did not go anywhere and I did not appear in public at any religious ceremony of Muharram month last year. In these mourning ceremonies, if you cry in public, folk say that she is crying for her infertility. If you avoid crying, they say, she is hard-hearted" (Participant 22).

## Discussion

Results of this study revealed that infertility is a source of social suffering for Iranian infertile women. This issue can fuel and exacerbate the many significant social problems in everyday life of couples. As the findings shown, infertility can be an underlying cause for violence (stigma, loss of social status and domestic physical violence), marital instability, social isolation and exclusion, relative deprivation, and social alienation.

Results of studies in Africa and Asia including Pakistan, Kuwait, Turkey and Iran showed that infertile women by some means suffer from domestic physical violence, verbal violence and stigma by in-laws and people around them (31- 37). In a study conducted in India, 70% of women had experienced domestic physical violence, while 20% reported severe physical violence due to infertility. The evidence demonstrated that rate of physical violence in India is comparatively higher than other developing countries (4). The prevalence of domestic violence among Iranian infertile women has been reported by Ardabily et al. as 61% with the majority being psychological violence in origin. Moreover, the mentioned-study has also indicated that 14% of the women suffered from physical violence due to infertility (36). The prevalence of physical violence in Iran is lower than other regional Muslim countries like Pakistan. In Pakistan, 23% of infertile women reported physical violence (38). In our study, just two of the participants experienced physical violence by their husbands. Despite this fact, most of the infertile women in this study faced the psychological violence, largely by their husbands and relatives. This is consistent with the findings of an earlier study conducted in Nigeria (32). As the study showed that infertile women in Nigeria experienced various forms of domestic violence including psychological torture, physical and verbal abuse and ridicule. Likewise, the participants of our study reported psychological violence in multiple forms such as discrimination, shame and humiliation. The present study is in agreement with the studies by Abbasi-Shavazi et al. (14) and Inhorn conducted in Egypt, indicating that gamete donation method may lead to stigma in the communities. Therefore, infertile couples usually try to hide their treatment method from others fearing that their children would not be accepted as their biological children (39).

In addition, results of a qualitative study in infertile women with secondary infertility in Pakistan confirmed that infertility is a main factor influencing marital instability, particularly for women who had no living child. Participants of the focus group discussions (FGD) in this study altogether agreed that the infertile women are being threatened with divorce and remarriage by their husbands and the relatives (33). Similarly, Wiersema et al. in a mixed methods (MM) study in South Vietnam showed that women with the unexplained infertility felt that their marriage is threatened, and some of them were afraid that their husbands will leave them (40).

A qualitative study in Sweden also addressed that about half of the participants separated from their husbands, and in all cases, the men had left the women (41). In our study, none of the participants were threatened with divorce due to infertility, whereas the main factors increasing the likelihood of divorce were social and psychological consequences of infertility, such as disagreement of women about remarriage of their husbands or potential involvement of women with socio-psychological consequences of infertility, leading to husbands’ frustration. This study also revealed that likelihood of divorce was increased by positive encouragement and pressure of in-laws for remarriage of husband. Perhaps this issue is due to the taboo surrounding infertility problem in an Islamic country. Additionally, like many developing countries, remarriage is considered as a man’s right when there are barriers to having a baby. Molock (42) has showed that different cultures have the following three ways to deal with infertility: i. some accept social solutions, such as divorce, polygamy and the adoption, ii. many use medical techniques and medical plants, while iii. in some cultures, resorting to spiritual people and pilgrimage places are chosen.

Additionally, results of mixed methods and qualitative studies have also showed that a large majority of participants in these studies experienced social pressure to have a baby (33, 37, 40). In the same context, a good number of the participated infertile women in South Vietnam stated that they preferred to stay at home and avoided contacting and dealing with children as a copping strategy for managing their challenging feelings and emotions due to infertility (40). Results of a qualitative study in Sweden showed that nearly all of the infertile women had experienced the social isolation (41). Some infertile couples isolate themselves from social activities due to fear of being confronted with pregnant women. They have a sense of “strained social interaction” and they have to have a smiling face when they confront other pregnant women (18). Likewise, our participants of our qualitative study also found the best way to deal with the psychological and social consequences of infertility was to avoid certain ceremonies or kids and pregnant women.

A closer look at the literature has suggested that in some countries, infertile individuals are considered as a source of misfortune, leading to be widely rejected by the people (4, 34). In some developing countries like Pakistan infertile women, as a carrier of bad luck, are excluded from or at least not welcomed to important social events, festivals, ceremonies and birthday. People look at them with hate and dislike and are afraid to allow their children to touch them (37, 12). These findings were not experienced by our participants. They related their social exclusion to the fact that in absence of children, relatives and friends paid little attention to them and minimized social interactions with them. Authors acknowledge that the movement towards modernization in Iran may lead people to overlook cultural values and ideals.

**Fig 1:**
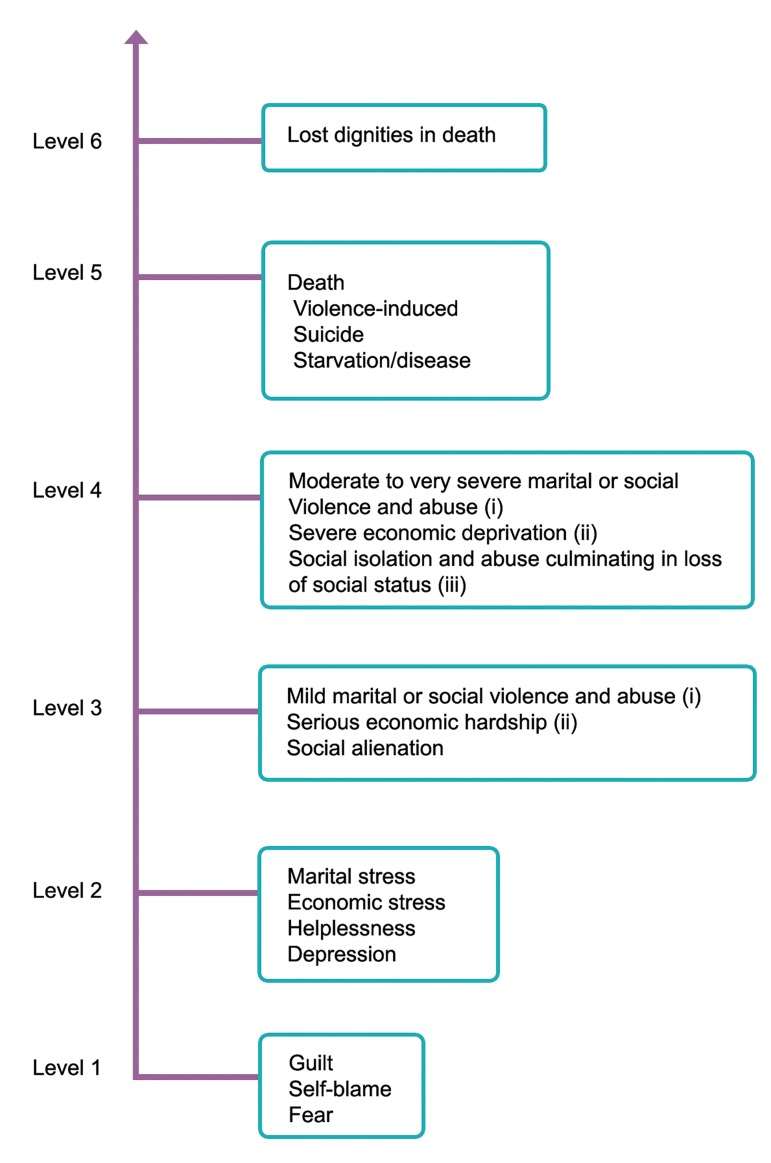
Continuum of the consequences of infertility [Daar and Merali (4)].

The World Health Organization (WHO) has presented the consequences of infertility for the infertile people using a diagram shown in figure 1. As this diagram reported, the consequences of infertility in the developed countries can rarely go beyond the level two of the spectrum, while in the developing side, at least in Asia and Africa, the consequences of infertility are infrequently as mild as the level three (4).

In this view, participants experienced all social consequences of infertility at the level three. As shown in the fig 1, the social alienation, as one of the five main categories of this study, is included in the level three of the spectrum.

Our findings showed that serious economic problems for most participants were the result of medical expenses, while in other studies, infertility alone leads to serious economic deprivation for infertile female by the husband or inlaws (43, 44). A mixed method study in Rwanda has revealed that if women are unable to become pregnant, husbands have a right to refuse to buy food and clothing for them. The in-laws also refuse to give any inheritance to infertile woman in the death of her husband (43).

Despite many similarities in social consequences of infertility in the developing countries including Muslim societies, the evidence of this study showed that there are also significant differences in type and extent of the social consequences of infertility. As our study revealed, these difference may be attributed to the transitional nature of Iranian society in terms of demography, health and sociocultural arrangement. As Dyer (45) also observed, regardless of the differences in sociocultural backgrounds, negative social consequences for infertility in different regions are similar in most cases.

However, there is a significant difference between the experience of infertility in developing and developed countries (2). In developing countries, ability to conceive is so central to couples’ identities, especially for women (4). In developed societies, voluntary childlessness is viewed as a more viable and legitimate option and women without children are often presumed to be voluntarily childfree (2) Therefore, through rejecting and resisting discourses that equalize femininity with motherhood, childless women create new discourses that can subvert and transform constructions of femininity (46). In cultures in which there is no concept of voluntary childfree status, it is impossible to hide infertility. Distress of infertility, therefore, is likely to be greater in developing countries (31). The researchers recognize the necessity of selecting their case studies from women with different socio-economic levels since it is an influential parameter on the complications following the treatment. However, since most advantaged infertile individuals use private infertility centers, and these centers denied researchers’ request for interviews, the study did not include women from this group. The researchers tried to partially overcome this limitation with adding some cases to the study by picking women who used public clinics, but they were from higher income families. It is noteworthy to point out that we tried to encourage the infertile women in sharing their experiments by explaining the goals of the study.

## Conclusion

The results of this study showed that infertility is more than a natural and biomedical entity in the marital life. It has complex interactions with social relationships, expectations and needs, affecting widely everyday life of infertile women. On this basis, there is a need for facilitation and prioritization of infertility treatment to prepare plans for empowering infertile women in various aspects of their lives. Our study showed that the different categories of the socio-emotional consequences of infertility. Increasing public awareness about the infertility and its multiple consequences, and adherence to the sexual and reproductive rights of women can be helpful in improving women’s health and stability of family life in the context of Iranian society.

This study also indicates that biomedical interventions are not sufficient to manage the issue of infertility, and it is required to pay further attention to its hidden consequences and manifest effects in all aspects. It is also needed to understand and to manage properly the issue of infertility in the context of societies in transition; therefore, we suggest that the professional social workers provide care facility in order to help infertile people and to increase awareness about consequences of infertility in the social system in which infertile people are living (34). Furthermore, professions like social workers can support the rights and needs of infertile people as a means to development planning by policy-makers, so that the infertility can be looked upon as a biopsychosocial phenomenon.
